# Regulatory Effect of Astragalus Polysaccharides on Intestinal Intraepithelial γδT Cells of Tumor Bearing Mice

**DOI:** 10.3390/molecules190915224

**Published:** 2014-09-23

**Authors:** Shuyu Sun, Kang Zheng, Hongyan Zhao, Cheng Lu, Biao Liu, Changyuan Yu, Ge Zhang, Zhaoxiang Bian, Aiping Lu, Xiaojuan He

**Affiliations:** 1 Institute of Basic Research in Clinical Medicine, China Academy of Chinese Medical Sciences, Beijing 100700, China; E-Mails: ssy_1015@163.com (S.S.); lv_cheng0816@163.com (C.L.); lvap@mail.cintcm.ac.cn (A.L.); 2 College of Life Science and Technology, Beijing University of Chemical Technology, Beijing 100029, China; E-Mail: yuchangy@sohu.com; 3 School of Life Science and Engineering, Southwest Jiaotong University, Chengdu 611756, China; E-Mail: zhengkang110@gmail.com; 4 Institute of Basic Theory, China Academy of Chinese Medical Sciences, Beijing 100700, China; E-Mail: zhaohongyan1997@163.com; 5 School of Chinese Medicine, Hong Kong Baptist University, Hong Kong, China; E-Mails: 13261526031@163.com (B.L.); zhanggehk@gmail.com (G.Z.); bianzxiang@gmail.com (Z.B.)

**Keywords:** astragalus polysaccharides, enteric mucosa, γδT cells, tumor

## Abstract

Astragalus polysaccharides (APS) possess multiple immunomodulatory activities. Due to its high molecular weight, orally administration of APS is not easily absorbed into the blood stream, and how APS exerts its capacity *in vivo* is still not well elucidated. We assume that enteric mucosal immune response might trigger the immune regulation of APS, and our previous studies demonstrated that APS had regulatory activity on intraepithelial lymphocytes (IELs). Therefore, this study aimed to investigate the functions of APS on intestinal intraepithelial γδT cells, a major subset in IELs and an essential component of maintaining homeostasis and immune regulation in enteric mucosa. Results showed that APS could promote proliferation and function of intestinal intraepithelial γδT cells *in vitro*, the IFN-γ, FasL and GrB mRNA levels in γδT cells were all significantly increased. Moreover, APS also improved the activity of intestinal intraepithelial γδT cells *in vivo*, as cytokines production and cytotoxicity of γδT cells were all remarkably improved in tumor-bearing mice treated with APS. In addition, the levels of TNF-α and IFN-γ were significantly increased, whereas the levels of IL-10 and TGF-β were significantly decreased in tumor-bearing mice treated with APS. In conclusion, this study demonstrated that APS could improve proliferation and function of intestinal intraepithelial γδT cells, which might an important pathway for immunomodulation of APS in cancer therapy.

## 1. Introduction

Astragalus polysaccharides (APS), the main efficacious principles extracted from *Astragalus membranaceus* (a traditional Chinese medicinal herb), have been proved to possess multiple immunomodulatory functions, including inhibiting the proliferation of CD4+CD25+ regulatory T (Treg) cells [1], promoting the maturation of dendritic cells (DCs) [2], enhancing the cytostatic activity of macrophages [3], regulating the imbalance of Thl/Th2 subgroups, and differentiation of the erythroid lineage [4,5], *etc.* It is regarded as a hopeful adjuvant to cancer therapy [6,7]. However, due to its high molecular weight, orally administration of APS is not easily absorbed into the blood stream directly for functional activity. Until now, how APS exerts its capacity *in vivo* is still not well known.

Previous studies have indicated that nutrients, including saccharides, are absorbed in the intestine [8]. As the intestine is an important immune organ consisting of a complex cellular network [9], we, therefore, assume that enteric mucosal immune response might trigger the immune regulation of APS. The intestinal mucosal immune system is composed of three major lymphoid areas: the lamina propria (LP), the intraepithelial compartment (contains intraepithelial lymphocytes (IELs)), and lymphoid nodules [10]. As IELs are located above the basement membrane and directly in contact with enterocytes and antigens, they play key roles in resisting invading pathogens and balancing protective immunity [11]. Generally, many traditional Chinese Medicines (TCM) are used by orally, they, therefore, may have extensive interactions with these cells. Research has demonstrated that some orally administrated TCMs could regulate the activities of IELs [12,13]. Our previous studies also indicated that APS had regulatory activity on IELs, as some cancer-related gene expressions were up-regulated or down-regulated in immune-suppressed mice after being orally administrated with APS [14].

However, IELs are extremely heterogeneous, and the various IEL subsets are distributed differently in the epithelium of the intestine [11]. γδT cells, constituting up to 60% of small intestinal IELs, are a significant proportion of IELs [15]. Emerging evidence showed that γδT cells from IELs played a crucial role in mucosal repair, regaining homeostasis, and possibly even tumor surveillance [16]. Further, mucosal γδT cells can regulate local and systemic immune responses [17]. After aerosol challenge with ovalbumin, γδT cell deficient mice exhibited a significantly decreased migration of B cells and natural killer cells to airways, and reduced levels of allergen-specific IgG and IgA in bronchoalveolar lavage fluid. Additionally, systemic allergen-specific IgE response was also defective [18]. Intraepithelial γδT cells, with other intestinal tract cells, have multiple cross-talks. Intestinal intraepithelial γδT cells can directly interact with neighboring epithelial cells and regulate mucosal IgA production [19,20]. They can also migrate actively within the intraepithelial compartment, and into the lamina propria, to establish extensive contacts with epithelial cells [21]. Therefore, in this study, we further investigated the effect of APS on γδT cells from small intestinal IELs.

## 2. Results and Discussion

### 2.1. APS Promote Proliferation and Function of Intestinal Intraepithelial γδT Cells in Vitro

**Figure 1:**
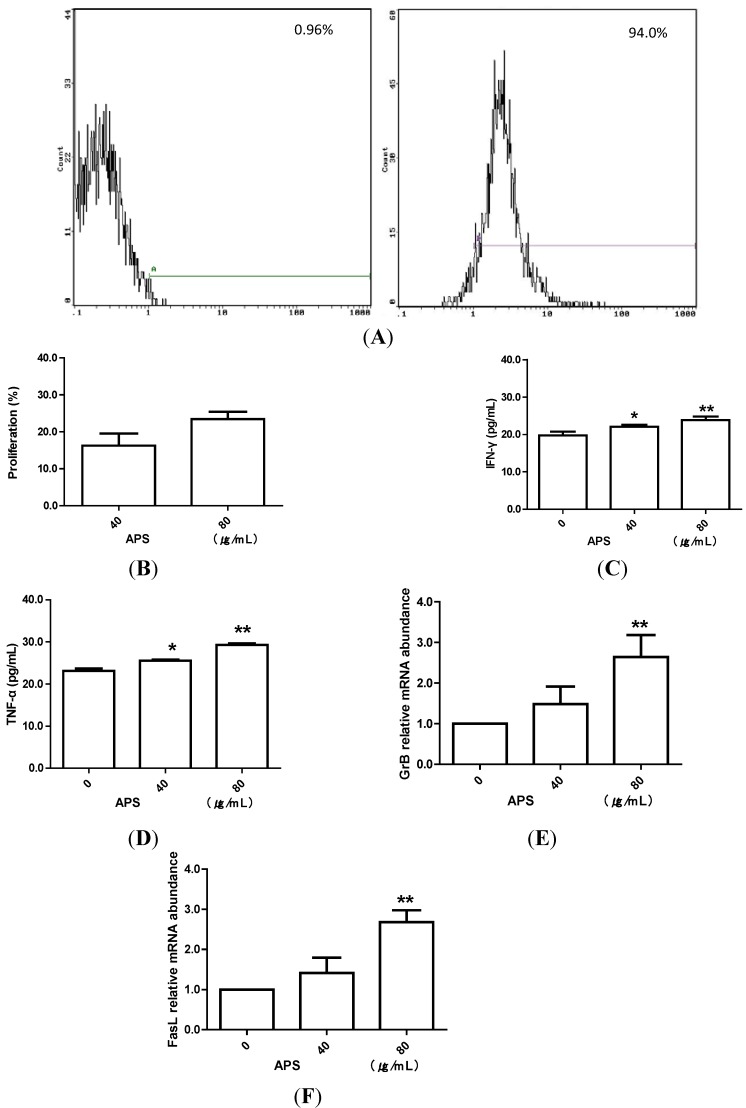
APS stimulates mice intestinal intraepithelial γδT cells *in vitro*. (**A**) A representative flow cytometric results of purified intestinal intraepithelial γδT cells. (**B**) CCK-8 assay to measure the proliferation of γδT cells treated with different concentrations of APS. (**C**,**D**) ELISA assay to detect the concentration of IFN-γ and TNF-α in cultivated supernatants of γδT cells treated by APS. * *P* < 0.05, *vs.* control; ** *P* < 0.01, *vs.* control. (**E**,**F**) Real-time PCR assay to detect the mRNA levels of FasL and GrB in γδT cells treated by APS. ** *P* < 0.01, *vs.* control.

To detect the effect of APS on γδT cells *in vitro*, we firstly isolated intestinal intraepithelial γδT cells by magnetic activated cell sorting. The purity of γδT cells was 94% (Figure 1A). Then, these purified γδT cells were used for proliferation and function assay. We found that APS induced significant proliferation of intestinal intraepithelial γδT cells *in vitro* (Figure 1B). The levels of IFN-γ and TNF-α were remarkably improved in cultivated supernatants of γδT cells treated by APS when compared with those in controls (Figure 1C,D). Moreover, FasL and GrB mRNA levels in γδT cells were also significantly increased after treatment with APS (*P* < 0.01) (Figure 1E,F).

### 2.2. APS Inhibit Tumor Growth in S180 Sarcoma-Bearing Mice

**Figure 2:**
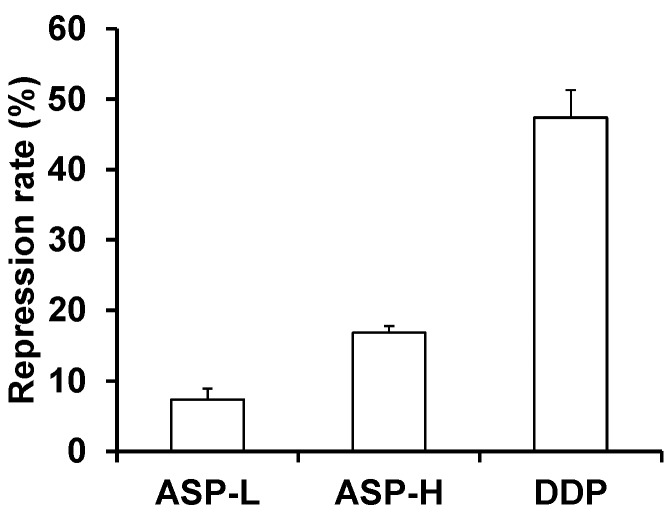
APS inhibits tumor growth in tumor bearing mice. The repression rate (%) = (mean weight of tumor of model control group − mean weight of tumor of drug-treatment group)/mean weight of tumor of model control group × 100%.

Tumor weights were detected in all tumor-bearing mice. The tumor weight in all of the treatment groups significantly decreased compared with the untreated control group. The tumor inhibition rate in the mice of the APS-L, APS-H, and DDP groups reached 7.44%, 16.76%, and 47.12%, respectively. These results demonstrated that APS treatment promoted tumor remission (Figure 2).

### 2.3. APS Improve Activity of Intestinal Intraepithelial γδT Cells from Tumor-Bearing Mice

**Figure 3:**
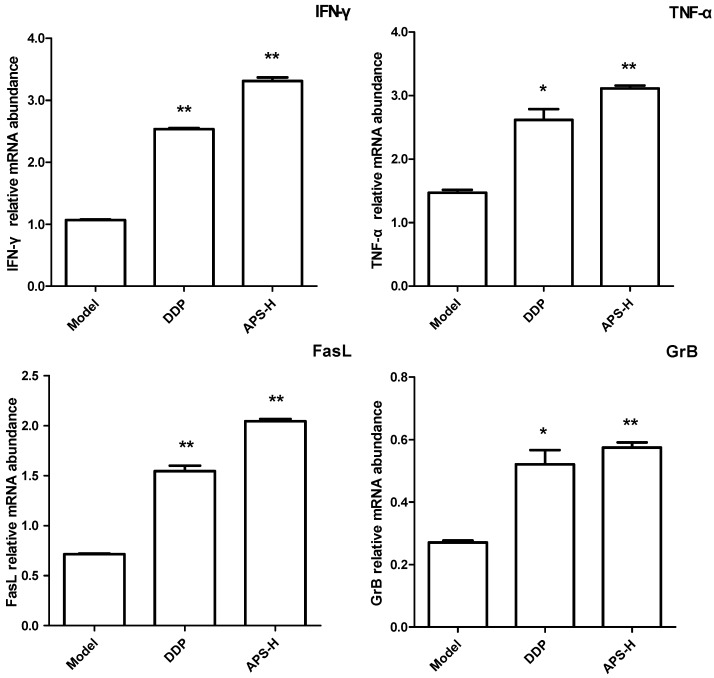
APS stimulates intestinal intraepithelial γδT cells from tumor-bearing mice. Real-time PCR assay to detect the mRNA levels of IFN-γ, TNF-α, FasL, and GrB in intestinal intraepithelial γδT cells separated from tumor-bearing mice. * *P* < 0.05, *vs.* model group; ** *P* < 0.01, *vs.* model group.

To detect the effect of APS on cytokine production and cytotoxicity of γδT cells *in vivo*, we examined TNF-α, IFN-γ, FasL and GrB mRNA levels of intestinal intraepithelial γδT cells in S180 tumor bearing mice treated with APS. Because of the very large workload and limited time, we only chose the model group, DDP group and APS high dose (APS-H) group. We found that the levels of these four factors were all significantly increased in tumor-bearing mice treated with a high dose of APS when compared with those in the model group (*P* < 0.01) (Figure 3).

### 2.4. APS Restore Th1/Th2 Cytokines Balance in Serum of Tumor-Bearing Mice

**Figure 4:**
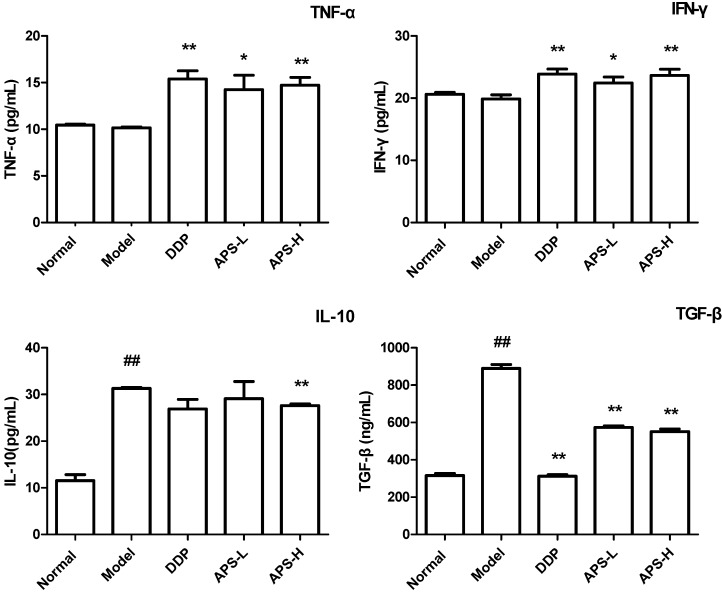
APS regulates Th1/Th2 cytokines balance in tumor-bearing mice. Expression of cytokines TNF-α, IFN-γ, IL-10 and TGF-β in serum were determined using ELISA. ^##^ *P* < 0.05, *vs.* normal group; * *P* < 0.05, *vs.* model group; ** *P* < 0.01, *vs.* model group.

To determine the Th1 and Th2 cytokine production in tumor-bearing mice, expressions of TNF-α, IFN-γ, IL-10 and TGF-β in serum were measured. The data demonstrated that the levels of TNF-α and IFN-γ were significantly increased (*P* < 0.01), whereas the levels of IL-10 and TGF-β were significantly decreased in tumor-bearing mice treated with APS when compared with those in the model group (*P* < 0.01) (Figure 4).

### 2.5. Discussion

In this study, we demonstrated that Astragalus polysaccharides (APS), one of the main active components in *Astragalus membranaceus*, played an immunomodulatory role partly through regulating the function of intestinal intraepithelial γδT cells.

γδT cells, bridging innate and adaptive immunity, are a minor population of T cells that express the TCRγδ chains. Although less in the periphery, they are abundant in epithelial and mucosal tissues [22]. Intestinal intraepithelial γδT cells are regarded as an essential component of maintaining homeostasis and immune regulation [17]. They have an activated phenotype, characterized by CD69 expression and increased cell size compared with systemic T lymphocytes. Additionally, the majority of intestinal intraepithelial γδT cells express the CD8αα homodimer. On the other hand, CD8αα+ γδT cells in IEL showed high basal calcium levels in contrast to systemic γδT cells [23]. After being activated, they can, not only secrete several cytokines, such as TNF-α and IFN-γ, but also possess cytotoxic capabilities, which contribute to protective immunity [11]. Therefore, in this study, we further investigated the role of APS on intestinal intraepithelial γδT cells. Our data demonstrated that APS promoted proliferation and protective cytokines production of intestinal intraepithelial γδT cells *in vitro*. In addition, γδT cells can utilize FasL-mediated and perforin/granzymes-based cell cytotoxicity pathways in exerting tumor cells and infected cells death [24,25], thus, we then detected FasL and GrB mRNA levels in γδT cells after APS administration. We found that the mRNA levels of FasL and GrB were significantly increased in intestinal intraepithelial γδT cells treated with APS, indicating that APS improved γδT cells’ cytotoxic activities. Furthermore, oral administration of APS could also improve the function of intestinal intraepithelial γδT cells in tumor-bearing mice, as protective cytokines production and cytotoxic potential were all promoted by APS treatment. All these data showed that APS could improve activities of intestinal intraepithelial γδT cells *in vitro* and *in vivo*.

To detect the effect of APS on systemic immune responses, we subsequently examined the expression of Th1/Th2 cytokines in serum of tumor-bearing mice. We chose TNF-α, IFN-γ, IL-10, and TGF-β due to their important roles in tumor immunity [26,27]. The data demonstrated that APS restored Th1/Th2 cytokines balance in serum. Our other studies also showed that orally administrated APS reduced the proportion of Treg cells and Gr-1+CD11b+ myeloid derived suppressor cells in tumor bearing mice (data not shown). All these results demonstrated that orally administration of APS could promote anti-tumor systemic immune responses.

The importance of mucosal immunology lies in the interaction between the mucosal response and the systemic immune response. Several studies have demonstrated that stimulation of the mucosal immune response can result in the production of protective B and T cells in both mucosal and systemic environments [28,29]. There exists extensive cross-talk between intraepithelial γδT cells and other intestinal cells, as well as the enteric mucosal immune system and systemic counterparts. Intestinal intraepithelial γδT cells can not only interact with neighboring epithelial cells [19,20], but also migrate rapidly and extensively within the intraepithelial compartment and into the lamina propria to survey large areas of villous epithelium [21]. On the other hand, CD103+ DCs in the intestinal lamina propria (LP) and mesenteric lymph node (MLN) can induce FoxP3 Treg cells differentiation [30,31]. Intestinal epithelial cells (IECs) can also function as nonprofessional antigen presenting cells and regulate T cell responses [32]. All these interactions constitute a very large and complex functional regulation network. Once stimulated, they will soon produce chain reactions. Previous studies showed that oral administration of glutamine and probiotics could ameliorate the severity of polymicrobial sepsis and colitis through regulating γδT cells in IELs, respectively [33,34]. Though our present study found APS could regulate the activities of intestinal intraepithelial γδT cells, how APS regulates the systemic specific immune responses through intestinal intraepithelial γδT cells still need further studies.

The intestinal mucosal immune system is composed of variety of cells. In addition to γδT cells in IELs, other studies have showed that APS could regulate inflammatory cytokines expression of intestinal epithelial cells [35]. We also found that APS increased the production of IgA and the number of Peyer’s pathes (PPs) in enteric mucosa in tumor-bearing mice [36]. These indicated that, in addition to intestinal intraepithelial γδT cells, APS might have multiple effects on the enteric mucosal immune system. How APS influences the variety of cells in intestine and the interactions of these cells need more studies.

## 3. Experimental Section

### 3.1. Experimental Animals and Cell Lines

Male BALB/c mice, 6–8 weeks of age, were purchased from the National Institutes for Food and Drug Control (Beijing, China) (Rodent license No. SCXK 2009-0017). The experimental procedures were reviewed and approved by the Animal Care and Use Committee in the China Academy of Chinese Medical Sciences before animal experiments were performed. Mouse sarcoma S180 cell lines were obtained from Cell Resource Center at the Institute of Basic Medical Sciences, Chinese Academy of Medical Sciences (Beijing, China) and cultured in RPMI-1640 medium (Gibco, Grand Island, NY, USA) supplemented with 10% fetal calf serum (FCS) (Gibco, Grand Island, NY, USA).

### 3.2. Drug

Astragalus polysaccharide (APS) with a purity above 98% was purchased from Pharmagenesis, Inc. (Redwood City, CA, USA) (batch No. 121008). There was no detectable level of endotoxin (<0.10 endotoxin units/mL) in the APS samples using an Endospecy assay.

### 3.3. Isolation of Intestinal Intraepithelial γδT Cells

Isolation of γδT cells from the intraepithelial lymphocytes (IELs) was performed as described previously [37,38,39]. In brief, the intestine from the duodenum to the ileocecal junction was removed and flushed of fecal material. The mesentery was removed, and the intestine was cut into pieces about 10 cm long, and then Peyer’s patches were cut out and discarded. The pieces were placed in 50 mL centrifuge tubes at 0 °C for 60 min. Then the pieces were digested for 30 min in PBS containing 1 mM EDTA, 1 mM DTT, 2% FBS by shaking at 37 °C. The pieces were opened longitudinally, and the eluted cells were collected and passed through a nylon mesh strainer to remove undigested tissue pieces. After centrifugation, cells were resuspended in 30% Percoll. The IELs were subsequently separated from epithelial cells by two centrifugations through a 40%/70% Percoll gradient at 450× *g* for 30 min. Thereafter, PE-conjugated anti-TCRδ-chain (GL3) antibody was added in the IEL suspension to purify intestinal intraepithelial γδT cells by magnetically activated cell sorting (Miltenyi Biotec., Bergisch Gladbach, Germany). The purity of the γδT cells was determined by flow cytometry. Matched PE conjugated isotype was used as a negative control.

### 3.4. γδT Cells Preparation and Treatment

Purified γδT cells (1 × 10^6^ cells/well) were cultured in RPMI 1640 medium in 48-well flat bottom plates (Corning, Glendale, AZ, USA) at 37 °C, 5% CO_2_, supplemented with 10% fetal calf serum (FCS). Then, different concentrations of APS (0, 40 and 80 μg/mL) were added, and cells were incubated for 3 h. The cultured supernatants were collected and stored at −80 °C for ELISA assay of cytokines concentrations. Cells were collected for real-time PCR assay.

### 3.5. Cell Proliferation Assay

γδT cells were seeded on 96-well (3 × 10^5^ cells/well) cell culture plates (Corning, Glendale, AZ, USA). Then, different concentrations of APS (0, 40 and 80 μg/mL) were added. Six hours later, CCK-8 (10 μL/well) was added and incubated for an additional 4 h. The absorbance at 450 nm was subsequently measured with a microplate reader. The percentage of proliferation was calculated using the following formula: (T_A_− C_A_)/C_A_ × 100%. T_A_ = Absorbance of cells for test, C_A_ = Absorbance of cells for control.

### 3.6. Animal Treatment

Mice were subcutaneously implanted with S180 cells in the right axillary region. Then these mice were randomly grouped as follows (n = 10 for each group): model group, diamminedichloroplatinum (DDP) group, APS low dose (APS-L) group, and APS high dose (APS-H) group. Another ten normal mice were used as normal control. After 24 h, mice in the normal group, model group, and APS group were orally administrated with different drugs at a volume of 200 μL/mice for 14 days. Normal group: saline; model group: saline; APS-L group: 150 mg/kg/d APS; APS-H group: 300 mg/kg/d APS. Mice in the DDP group were given 2 mg/kg DDP by intraperitoneal injection every other day [7,40,41]. After fourteen days of treatment, mice were sacrificed. Vein blood was obtained and serum was collected by centrifugation at 3000 r/min for 20 min. Intestinal intraepithelial γδT cells were isolated as described above. The collected serum and γδT cells were stored for further analysis.

### 3.7. ELISA for Detection of Cytokines Production in Cultured Supernatants and Mice Sera

Concentrations of IFN-γ and TNF-α in cell cultured supernatants and concentrations of TNF-α, IFN-γ, IL-10, and TGF-β in mice sera were determined by means of commercially available ELISA kits (eBioscience, San Diego, CA, USA) according to the manufacturers’ instructions.

### 3.8. Real Time-PCR for Detection of TNF-α, IFN-γ, FasL and Gram B Expression in Intestinal Intraepithelial γδT Cells

The levels of TNF-α, IFN-γ, FasL, and granzyme B (GrB) mRNA in γδT cells were analyzed by real-time PCR. Total RNA was extracted from γδT cells using the Ultrapure RNA kit (CWbio.Co., Beijing, China) following the manufacturer’s instructions. cDNA was synthesized from the total RNA using the HiFi-MMLVcDNA kit (CWbio.Co., Beijing, China). Real-Time PCR was performed using the RealSuper Mixture (with Rox) kit (CWbio.Co., Beijing, China). GAPDH was used as an invariant housekeeping internal control gene. The primer sequences were as follows: TNF-α, forward: 5'-GAGAAGTTCCCAAATGGC-3', and reverse: 5'-ACTTGGTG GTTTGCTACG-3'; IFN-γ, forward: 5'-TAACTCAAGTGGCATAGATGTGGAAG-3', and reverse: 5'-GACGCTTATGTTGTTGCTGATGG-3'; FasL, forward: 5'-TGGCCTTGTGATCAACGAAACTG-3', and reverse: 5'-TTCAACCTCTTCTCCTCCATTAGCA-3'; GrB, forward: 5'-AAGCCAGGAGATGTGTGCTATGT-3' and reverse: 5'-AGCGTGTTT GAGTATTTGCCCATT-3'. Cycling conditions consisted of a denaturation step at 95 °C for 10 min, followed by 40 PCR cycles of 95 °C for 15 s and 60 °C for 60 s. Target genes were quantified using the 2-ΔΔCt method. The results were presented as relative levels of TNF-α, IFN-γ, FasL, and GrB mRNA.

### 3.9. Statistical Analysis

All data were analyzed using the SPSS 18.0 statistical package. The data were expressed as mean ± standard deviation (S.D.), and significant differences were assessed using a variance test. Results were considered statistically significant at the probability (*P*) values of <0.05 level.

## 4. Conclusions

In conclusion, this study demonstrated that APS could improve proliferation and function of intestinal intraepithelial γδT cells, which might be an important pathway for immunomodulation of APS in cancer therapy. However, more details on how APS regulates the immune system through intestinal intraepithelial γδT cells must be explored. 
